# Genetic and flow anomalies in congenital heart disease

**DOI:** 10.3934/genet.2016.3.157

**Published:** 2016-08-23

**Authors:** Sandra Rugonyi

**Affiliations:** 1 Department of Biomedical Engineering, Oregon Health & Science University, 3303 SW Bond Ave. M/C CH13B, Portland, OR 97239, USA

**Keywords:** blood flow, congenital heart defects, cardiac development

## Abstract

Congenital heart defects are the most common malformations in humans, affecting approximately 1% of newborn babies. While genetic causes of congenital heart disease have been studied, only less than 20% of human cases are clearly linked to genetic anomalies. The cause for the majority of the cases remains unknown. Heart formation is a finely orchestrated developmental process and slight disruptions of it can lead to severe malformations. Dysregulation of developmental processes leading to heart malformations are caused by genetic anomalies but also environmental factors including blood flow. Intra-cardiac blood flow dynamics plays a significant role regulating heart development and perturbations of blood flow lead to congenital heart defects in animal models. Defects that result from hemodynamic alterations, however, recapitulate those observed in human babies, even those due to genetic anomalies and toxic teratogen exposure. Because important cardiac developmental events, such as valve formation and septation, occur under blood flow conditions while the heart is pumping, blood flow regulation of cardiac formation might be a critical factor determining cardiac phenotype. The contribution of flow to cardiac phenotype, however, is frequently ignored. More research is needed to determine how blood flow influences cardiac development and the extent to which flow may determine cardiac phenotype.

## 1. Introduction

Congenital heart disease (CHD) occurs in at least one percent of newborn babies in the US and is responsible for more than 24% infant deaths each year [1–3]. Cardiac defects are the most common malformations in humans, and are the leading non-infectious cause of death in infants and children. Heart formation is a finely orchestrated biological process, but even slight perturbations in the developmental regulation of cardiac tissues may be sufficient to induce serious malformation. Family history of CHD accounts for a small proportion (~4%) of congenital heart defects [4], and while several genes have been associated with CHD, less than 20% of heart defect cases are linked to gene mutations [5]. This suggests a substantial role for non-genetic causes of CHD. Factors known to increase the risk of CHD in babies include maternal diabetes, obesity, and nutrition, all of which affect the *in utero* environment in which the embryo develops [6–8]. In addition, maternal exposure to smoking, alcohol and other teratogens are known to detrimentally influence the development of the baby and its heart [9,10]. Current research suggests that many cases of CHD have their origins in abnormal conditions during gestation, and these conditions are also associated with risks of cardiovascular disease later in life [11–15].

Among prenatal conditions that affect embryonic cardiac development, intracardiac blood flow has recently attracted a lot of attention. Cardio-genesis starts with the formation of a tubular heart, in which a primitive atrium, ventricle, and outflow tract are connected in series along the tube. Soon after formation, the tubular heart starts pumping blood, and intracardiac blood flow is established. As the heart continues to develop, cardiac septation and valve formation occur under blood flow conditions as the heart transforms from a tubular to a four-chambered structure, while pumping blood. Blood flow is essential for proper cardiac development [16], and abnormal blood flow conditions in animal models have been repeatedly shown to lead to cardiac defects similar to those in babies with CHD [17–21]. *In utero*, blood flow conditions in human babies can be detrimentally altered by genetic anomalies that induce changes in cardiac contraction and structure, and by environmental factors, including maternal exposure, placental anomalies, and inadequate vascularization of the vitelline circulation [14,22–24]. The interaction between cardiac tissue and blood flow determines biomechanical conditions to which the developing heart is exposed. Cardiac cells sense and respond to these biomechanical conditions through mechanotransduction mechanisms, which modulate genetic and epigenetic developmental programs [25–30]. Cardiac biomechanics, and cardiac blood flow in particular, are critical components of cardiac development, but unfortunately are currently not well understood nor completely characterized, and frequently ignored in the quest for causes of human CHD.

## 2. Congenital Heart Malformations

Babies with CHD present several phenotypes. Simple CHD cases involve relative `simple' heart defects such as ventricular septal defects (basically a hole in the cardiac wall that separates the right and left ventricles) or pulmonary stenosis (a narrowing of the pulmonary artery). Complex CHDs, on the other hand, consist of several simple defects, and are among the most severe cases of CHD typically requiring surgery. As examples, we will consider here two complex CHDs, tetralogy of Fallot and double outlet right ventricle, and we will discuss evidence that in both cases the defects could arise due to genetic anomalies but also due to other factors, such as altered blood flow in the absence of genetic or chromosomal abnormalities. In addition, we also discuss some of the regulatory genes and events that are associated with endocardial cushion formation, a process that occurs during early heart development and that influences later development of valves as well as septation, and can therefore be also associated with complex cardiac malformations.

Tetralogy of Fallot (TOF) is the most common complex CHD. TOF occurs in approximately 1 in 3,000 live births, or approximately 7 to 10% of CHD cases [31,32]. It is the most common type of cyanotic heart defect, and thus the most common cause of `blue baby' syndrome. TOF is characterized by four anatomical features: i) a ventricular septal defect (VSD) that mixes blood from the right and left ventricles; ii) obstructed flow of blood from the right ventricle to the lungs (pulmonary and subpulmonary stenosis); iii) displaced aorta (aorta dextroposition or overriding aorta) so that blood from both the right and left ventricles flow into the aorta (rather than just blood from the left ventricle); and iv) overtime TOF patients develop right ventricular hypertrophy. Severity of symptoms depends on the degree of pulmonary stenosis, and thus how much blood reaches the lungs for oxygenation. TOF is generally described as caused by anterior malalignment of the conal septum. Patient case studies and animal models suggest that a system of signaling pathways (e.g., JAG1, NKX2.5, TBX1) might be involved in pattern formation and vascular development giving rise to TOF. However, TOF might also arise as a result of other changes, as the specific genetic deficits or environmental exposures that lead to TOF remain unknown in the majority of patients.

Double outlet right ventricle (DORV), occurs in 1–3% of CHD cases [9]. DORV is a rare CHD that has a range of phenotypes. The main anatomical characteristic of DORV is that both the pulmonary artery and the aorta arise from the right ventricle, decreasing the amount of oxygenated blood delivered to the body. DORV is caused by failure of aortic translocation and regression of the subaortic conus. Most DORV defects are accompanied by other defects such as VSD, pulmonary stenosis, and transposition of the great arteries. Symptoms and urgency of treatment will depend on the specific anatomy of the heart. Studies have suggested involvement of certain gene pathways (e.g. CFC1, CSX, Cx40) in the genesis of DORV. However, most cases of DORV are not apparently the result of genetic anomalies and their causes remain unknown.

Endocardial cushions are formed early in embryonic development, during tubular stages of heart formation [33,34]. The endocardial cushions are thickenings of the tubular heart wall that develop in the atrio-ventricular canal, which connects the tubular primitive atrium and ventricle, and in the heart outflow tract [35,36]. In the tubular heart, endocardial cushions soon act as primitive valves, by closing the lumen upon myocardial contraction and limiting backflow of blood. Initially, endocardial cushions are mostly composed of extracellular matrix (cardiac jelly). An endocardial to mesenchymal transition (EMT) then occurs in which endocardial cells delaminate from the endocardium, invade the cardiac jelly, and differentiate into mesenchymal cells. EMT thus contributes to increased cell density of the endocardial cushions. The process of EMT, including the regulatory genes and growth factors involved (e.g. VEGF, NFATc1, Notch, TGF-β, snail, ErbB, BMP), have been well studied both in animal models and explant *in vitro* systems [33,34,37–41]. Proper development of endocardial cushions is required for normal development of valves and cardiac septation, and dysregulation of this process early on could lead to complex CHD and embryo-lethality [34].

## 3. Genetic Traits and Congenital Heart Disease

Finding genetic anomalies leading to congenital heart malformation has been a main focus of CHD research. From a basic scientific point of view, the quest goes beyond CHD, as a main goal is to understand how signaling pathways interact during development to give rise to a normal heart structure and how disruption of developmental processes, in contrast, lead to heart malformations. Main contributors of heart formation have been identified, and many temporal and spatial orchestration of events that leads to heart formation have been elucidated (e.g. [1,9,42–48]). Novel technologies and developments (e.g. high throughput sequencing) are bringing new tools to investigating the complexities of heart formation and malformation (e.g. [48–50]). From a clinical point of view, knowing the causes of CHD can lead to strategies for improved prognosis and treatment.

Cardiac formation is a complex spatio-temporal process. Some cardiac anomalies are the result of known mutations in developmental control genes [1]. For example, different genes and developmental pathways have been associated with TOF, DORV and defects of endocardial cushions (see Table 1). However, most CHD cases are not the result of single gene mutations. Thus researchers have also considered the effects of dysregulation of group of genes or signaling pathways in heart development perhaps through environmental interaction, and even epigenetic modifications [51]. Epigenetics here refer to changes in the chromatin structure that controls gene expression, and that can be inherited but also altered during development by programmed events as well as the *in utero* environment. An ever increasingly complex picture of cardiac development and regulation is emerging that requires consideration of several factors to explain normal and abnormal cardiac formation.

Despite extensive research into identifying genetic anomalies leading to CHD, most human cases (including the majority of TOF and DORV cases) still remain unexplained. Because cardiac development is a complex interaction between genetic programs and environmental effects, research to understand the causes of CHD naturally lead to studies about teratogen influences on heart development.

## 4. Environmental Exposures and Heart Formation

Environmental factors affect the *in utero* environment in which the baby develops and thus can affect heart formation. These factors include teratogens to which the mother is exposed, including certain drugs and medications, that transport through the placenta into the baby circulation exposing the baby; and viral infections [54]. More subtle environmental factors involve mother nutrition, obesity status and diabetes, as they affect transport through the placenta into the baby and the baby access to nutrients [6–8]. For example, diabetes increases the risk of offspring heart defects by 5 fold [55,56], presumably due to toxic effects of hyperglycemia on the development of the baby (with excess glucose transported from the mother to the baby through the placenta). Cushion formation during EMT has been shown to be affected by both hypoxia and hyperglycemia [33], and studies showed an association between maternal diabetes and DORV [9,57]. Prenatal exposure to ethanol, retinoic acid or theophylline have also been reported in humans with DORV and TOF [9] (see Table 2).

## 5. Alterations in Blood Flow: A common link and more?

**Figure 1:**
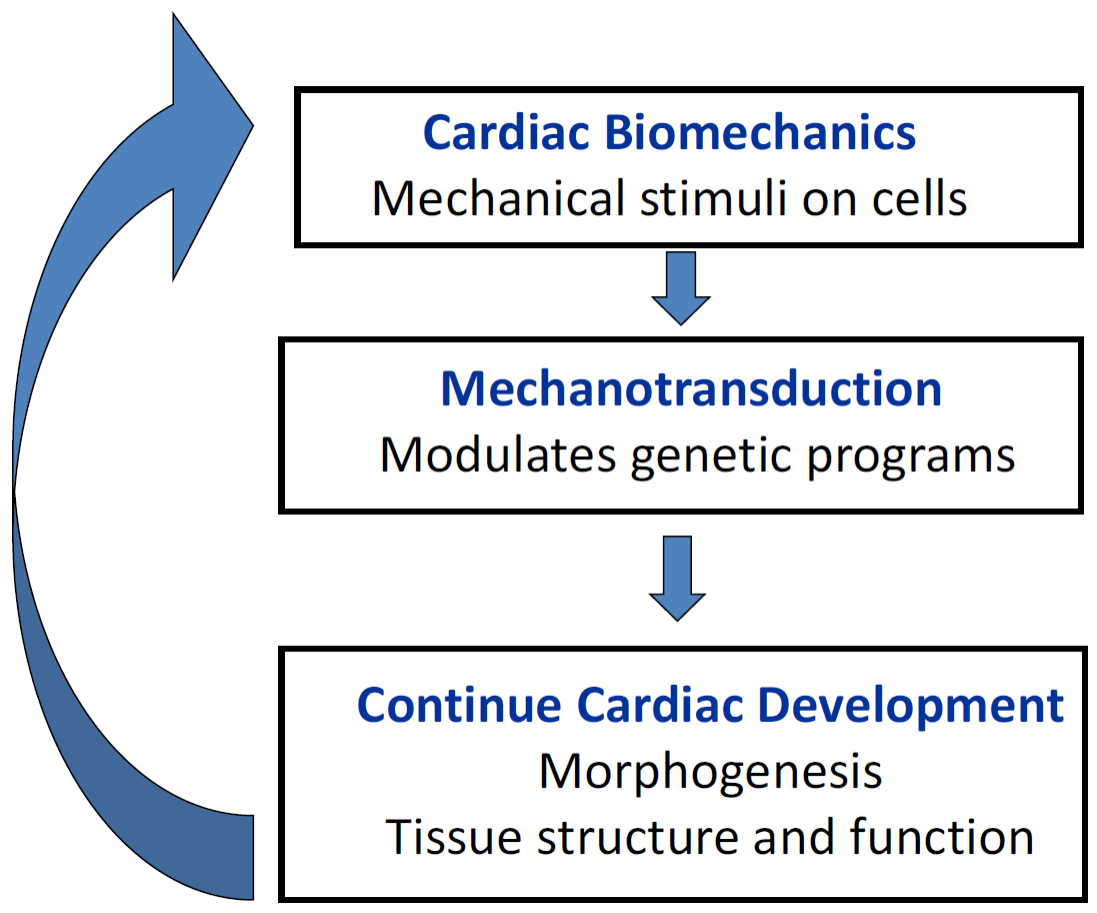
Schematics of hemodynamic feedback loop during cardiac formation. Cardiac biomechanics determine blood flow conditions in the developing cardiovascular system. Blood flow interacts with cardiac tissues, and this interaction exerts mechanical stimuli on cells. The cells sense and respond to these mechanical stimuli through mechanotransduction mechanisms that modulate heart development. Anomalous blood flow therefore leads to altered cardiac tissue structure through remodeling, and, as a consequence, altered tissue function. Eventually this dysregulation leads to altered morphogenesis, in turn affecting blood flow conditions.

Interactions between blood flow and cardiac tissues shape cardiac development. Blood flow is known to be an important regulator of cardiovascular development [16,17,21]. Cardiac cells sense the biomechanical heart environment (wall shear stresses, stretches) that results from the interaction of blood flow and cardiac tissues. In response to biomechanical stimuli, mechano-transduction mechanisms alter signaling pathways and cell behavior, modulating in these way pre-programmed genetic processes. This modulation, in turn, affects cardiac development, including cardiac morphogenesis and tissue composition, and therefore cardiac function (see Figure 1). Thus, in essence, blood flow acts as a feedback loop controlling cardiac development.

Altered blood flow in animal models leads to cardiac defects found in human babies with CHD. Blood flow has been manipulated in animal models to study how cardiovascular development is affected by hemodynamics, and the extent to which altered blood flow patterns lead to heart defects [16,19–21,59]. Several animal models have been used for this purpose, but the chicken embryo is perhaps the most popular model for altering blood flow conditions in early cardiac development. This is because chicken embryos are easy to access for flow manipulation and imaging inside the egg [35,60–63]. Surgical interventions that alter blood flow have been developed and used in chicken embryos, leading to a spectrum of congenital heart malformations, all of which resemble defects in human babies [17,19,21,29,30] (see Table 3). Moreover, some genes that respond to changes in blood flow conditions (including KLF2, ET1, NOS3) have been identified [28,29], and the initial consequences of altered blood flow on heart development are being elucidated [64,65].

During human development, blood flow conditions can be altered in different ways. Genetic anomalies and teratogens affect the way in which the heart and vasculature develops, and thus do lead in many cases to early alterations in blood flow conditions. Altered blood flow then, together with the original cause of disruption (gene anomaly, teratogen toxicity), continue to drive heart malformation. But blood flow can also be altered in babies due to placental anomalies, and anomalous development of the vitelline circulation, independently of the baby genetic makeup and toxic exposures. In these cases, CHD may arise without an apparent confounding cause.

In any case, altered blood flow patterns, once established, may in many cases be the main regulators of malformation. Unfortunately, it is very difficult to tease apart the effects of flow alone from those of other confounding factors. Further, researchers studying teratogen effects or genetic anomalies frequently disregard possible effects of flow and focus instead on phenotype and signaling pathways. The fact, however, that congenital heart defects, including ventricular septal defects, TOF, and DORV among others can be the result of genetic anomalies, teratogen toxicity, and abnormal blood flow independently, perhaps argues in favor of some common pathway leading to specific phenotypes. Altered blood flow conditions could be in many cases a common link among different exposures, and provide the means for malformations to develop.

## 6. Conclusions

Cardiac formation is the result of a complex interaction between genetic programs and environmental factors. Even subtle dysregulation of cardiac developmental processes, however, can lead to cardiac malformation. While the consequences of dysregulation in several genes and due to toxic exposures have been elucidated, more research is needed to fully understand how heart malformations develop, and more importantly how to prevent them or possibly reverse them. The critical role of blood flow patterns during heart development, in particular, needs to be further studied, both in the context of flow alterations and as a result of genetic anomalies and toxic exposures, to better understand the mechanisms by which congenital heart defects develop.

## Figures and Tables

**Table 1:** Gene anomalies associated with example cardiac defects.

Heart Defect Type	Associated genes	References
**TOF**	JAG1, NKX2.5, TBX1, NOTCH2, SALL4, MLL2, FOG2/ZFPM2, GATA6, HAND2, Cx40	[1,42,44,48,52,53]
**DORV**	CFC1, CSX, CRX, FOG2/ZFPM2, ZIC3, ET1,Cx40	[9,48,52]
**Defects of endocardial cushion**	TNNT2, CFK, Cx40,Cx46, Notch1, VEGF, NFATc1, Notch, TGF-β, snail, ErbB, BMP	[33,34,52]

**Table 2:** Environmental exposures associated with congenital heart defects.

Heart Defect Type	Environmental Exposure	References
**TOF**	ethanol, retinoic acid, theophylline, mother obesity	[7,9]
**DORV**	Maternal diabetes, ethanol, retinoic acid, theophylline	[9,57,58]
**Defects of endocardial cushion**	Hyperglycemia, hypoxia	[33]

**Table 3:** Altered blood flow conditions lead to congenital heart defects.

Heart Defect Type	Hemodynamic intervention model	References
**TOF**	Chicken (OTB)	*
**DORV**	Chicken (OTB, RVVL)	[20,21,52]
**Defects of endocardial cushion**	Cushion explants (altered flow); chicken (OTB)	[64–68]

OTB: Outflow tract banding also referred to as conotruncal banding; RVVL: right vitelline vein ligation;

* our unpublished results.
